# Comprehensive evaluation of sound absorption performance in porous concrete pavement with crushed stone base course

**DOI:** 10.1038/s41598-025-32128-1

**Published:** 2025-12-11

**Authors:** Yi Zhang, Hanbing Wang, Abul Khair, Jie Yang, Yunting Han

**Affiliations:** 1 School of Business, Fuyang Normal University, Anhui, 236037 China; 2 School of Civil Engineering, Chongqing Jiaotong University, Chongqing, 400074 China; 3 School of Chemistry and Chemical Engineering, Shanghai Jiaotong University, 800 Dongchuan Rd, Shanghai, 200240 China; 4 Key Laboratory of Road and Traffic Engineering of the Ministry of Education, College of Transportation, Tongji University, Shanghai, 201804 China; 5 College of Materials Science and Engineering, Zhejiang University of Technology, Zhejiang, 310014 China; 6 Composite Materials Research Institute, Zhejiang Huajiang Science and Technology Co., Ltd, Zhejiang, 311106 China; 7 Shanghai Research Institute of Building Sciences Co., Ltd, 75 Wanping South Road, Xuhui District, Shanghai, 200032 China

**Keywords:** Porous concrete, Crushed stone base, Sound absorption property, Sound absorption coefficient, Dominant frequency, Engineering, Materials science

## Abstract

This study comprehensively evaluates the sound absorption performance of porous concrete pavements with crushed stone base layers of varying thicknesses and particle sizes. The noise reduction potential of pavement structures was assessed using the standing wave tube method, and overall sound absorption was quantified via the full-frequency domain average sound absorption coefficient. Results indicate that the presence of a crushed stone base layer substantially enhances sound absorption, with thicker bases providing greater improvements. While particle size and surface layer type also influence absorption, their effects are markedly smaller than that of base course thickness. These findings suggest that optimizing base layer thickness, combined with high-porosity or fine-aggregate surface layers, can effectively improve the acoustic performance of porous concrete pavements, providing practical guidance for noise mitigation in road design.

## Introduction

With the accelerated pace of global urbanization, various types of vehicles have become the primary means of transportation for residents. However, the resulting traffic noise has emerged as a major environmental issue, significantly impairing the living comfort for residents along roadways^1–3^. Thus, solving the problem of traffci noise has emerged as ont only a primary technical difficulty for road professionals and city administractors, but also a pivotal challenge to the sucessful realization of China’s ecological civilization.

Tian and Niu^4^ (2008) categorized traffic noise sources into three types: vehicle system noise, aerodynamic noise, and tire-road contact noise, the latter being recognized as the predominant contributor of automotive noise. Tan et al.^5^ (2008) and Zhang et al.^6^ (2008) identified three main mechanisms for tire-road contact noise generation: tire vibration, air-pumping noise, and aerodynamic effects, which are fundamentally caused by solid vibrations, turbulent airflow, and unsteady pressure fluctuations. Research indicates that noise affecting the road sound environment primarily occurs below 1500 Hz, with the most significant concentration around 1000 Hz. Specifically, the spectral curve of tire-road contact noise exhibits a prominent peak within the 700–1300 Hz frequency range^5,7^.

To mitigate urban traffic noise, various noise-reducing pavements and sound barriers have been widely implemented in the construction of urban roads and related infrastructure^3^. Porous concrete is a functional pavement material composed of coarse aggregate, ordinary Portland cement, limited or no sand, additives (as required), and water. It is characterized by an extensive network of interconnected and semi-interconnected voids, which provides an excellent capacity for absorbing and mitigating tire-pavement noise^7^. Research indicates that porous concrete can reduce noise by 0.8-5dB compared to conventional concrete^8–10^. Consequently, porous concrete has been extensively applied in municipal road surfaces, paving bricks, noise barriers, and sound-absorbing panels, particularly in urban areas with stringent noise-control requirements^11–15^. Its large-scale application has demonstrated significant environmental and social benefits^1–3^.

The commonly used laboratory methods for evaluating the sound absorption performance of porous concrete can be categorized into five types: the standing wave tube method (impedance tube method), reverberation chamber method (simulated reverberation chamber), tire drop method, noise analyzer method, and PU in-situ surface impedance method^11,17–21^. Among these, existing research has predominantly focused on the laboratory-based standing wave tube approach^3^. Previous studies indicate that the sound absorption performance of porous concrete pavements is primarily influenced by pore characteristics, aggregate gradation, aggregate type, cement-aggregate ratio, admixtures, thickness, pavement structural composition, and environmental conditions such as temperature and humidity^1–3,22^.

It is generally accepted that the noise reduction performance of porous concrete is enhanced with its porosity^12,20–23^. However, some studies suggest that when porosity exceeds 25%, the sound absorption performance may decrease with further increases in porosity^10,17^. Research shows that porous concrete with different porosity levels demonstrates similar sound absorption capacity in the low-frequency range, but significant differences emerge in the mid-to-high frequency range^22^. As porosity increases, the sound absorption coefficients at the first and second dominant absorption frequencies (which occur when sufficient thickness is achieved) show noticeable improvement^22,23^. Galip et al.^15^.(2024) research indicates that pore size, pore area, and distribution pattern are the dominant factors affecting sound absorption performance. Similarly, Zhang^3^ (2021) study found that under identical porosity conditions, an increase in pore quantity of porous concrete can enhances its noise reduction. In contrast, other researchers argue that pore tortuosity and pore size have greater influence on sound absorption performance, while pore quantity has a relatively limited effect^3,24,25^.

For porous concrete with same porosity, it is generally observed that sound absorption performance improves with smaller aggregate sizes, indicating that finer aggregates are beneficial for noise reduction^3^. However, different studies have reported varying optimal aggregate size ranges^3,20,26,27^. The incorporation of recycled aggregates has shown minimal impact on noise reduction performance^24^. Notably, when slag is used as aggregate, porous concrete exhibits superior sound absorption compared with conventional virgin aggregates^2,28^. Wang and Zhao’s^16^ (2015) research found that porous concrete prepared with perlite aggregate achieved the highest sound absorption coefficient (0.66), outperforming those made with mineral slag (0.58) and ceramic particles (0.55). Other studies suggested that replacing virgin aggregates with rubber particles or lightweight aggregates can further enhance sound absorption capacity^2,29,30^. Aggregate gradation also plays a significant role in influencing sound absorption performance, although it does not alter the frequency or number of primary absorption peaks^3,31–34^. Additionally, while the sand-to-aggregate ratio affects overall sound performance, its influence on mid-to-low frequency absorption remains inconsistent across studies^27^.

Specimen thickness is a critical factor affecting the sound absorption performance of porous concrete, as it influences absorption characteristics across different frequency ranges^3,7^. Kim et al.^20^ (2018) and Zhang’s^3^(2021) research demonstrated that increasing thickness shifts the peak frequency of the sound absorption coefficient from higher to lower frequencies. Ni et al.^12^ (2014) and Jing et al.^35^ (2011) analyzed absorption curves of specimens with identical porosity but varying thicknesses and found that thicker specimens exhibited improved low-frequency absorption but reduced efficiency at higher frequencies. Lu’s^7^ (2013)research further demonstrated that thickness has the greatest impact on the dominant absorption frequency and suggested that optimal noise reduction is achieved when the absorption peak frequency of porous concrete matches the primary frequency band of traffic noise. In practice, an optimal thickness of approximately 4 cm is generally recommended for porous concrete pavement. However, considering vehicle type differences, Lu^7^ (2013) specifically recommended a thickness of 4 cm for passenger vehicle lanes and 6 cm for heavy-duty vehicle lanes. Moreover, several studies have shown that dual-layer structural designs can outperform single-layer structures of equivalent total thickness. In particular, a configuration consisting of a high-porosity surface layer combined with a low-porosity base layer has been recommended, although the optimal thickness distribution between layers remains a subject of debate^20,35^.

Previous research on the noise reduction performance of porous concrete pavament primarily focused on surface layer materials. However, in practical engineering applications, porous concrete pavements is often constructed in the form of a porous concrete surface layer with a gravel aggregate base course.Whether the presence of a gravel base course affects the sound absorption performance of porous concrte pavament has not yet been addressed by scholars.

The objective of this study is to utilize the standing wave tube method to inverstigate the sound absorption performance of porous concrete pavaments with a gravel aggregate base course. The research aims to elucidate the effects of key variables-including the aggregate size of the crushed stone base (2.36 ~ 4.75 mm, 4.75 ~ 9.5 mm, and 9.5 ~ 13.2 mm), the thickness of the crused stone base layer (4 cm, 7 cm, and 10 cm), and the the type of porous concrete surface material (with variations in aggreage size and cement to aggregate ration)- on the acoustic performance of the composite porous concrete pavament structure. The ultimate goal is to establish a theoretical basis for enhancing the noise reduction efficiency of this sustainable pavement structure.

## Materials and methods

### Materials

In this study, both the aggregates used for preparing porous concrete and the crushed stone base course were basalt aggregates sourced from Zhejiang Province, China. The basic physical properties of the coarse (9.5 ~ 13.2 mm), medium (4.75 ~ 9.5 mm) and fine (2.36 ~ 4.75) basalt aggregates commonly used in the production of porous concrete mixer are presented in Table 1. The cement used was Conch brand Grade 42.5 ordinary Portland cement, produced in Taicang, Jiangsu Province, China, with its technical specifications listed in Table 2. Laboratory tap water was used for mixing.

**Table 1:** Basic physical properties of the basalt aggregate.

Aggregate type	Aggregate Size (mm)	Density (g/cm^3)^	Bulk Density (g/cm^3)^	Porosity (%)	Needle-content (%)	Crushing value (%)
Basalt	2.36–4.75	2.899	1.649	42.3	-	20.20
	4.75–9.5	2.906	1.697	41.5	6.54	11.60
	9.5–13.2	2.944	1.741	42.3	2.39	10.30

**Table 2:** The technical specifications of the P.O 42.5 cement.

Density (g/cm^3^)	Specific surface area (m^2^/kg)	Setting time (min)		Compressive strength (MPa)		Flexural strength (MPa)	
		Initial	Final	3d	28d	3d	28d
3.15	360	175	234	27.5	49.0	5.5	8.0

### Mix proportions and sample preparation

This study primarily investigates the influence of aggregate particle size and thickness in the crushed stone base course on the sound absorption performance of porous concrete pavement structures. Accordingly, a relatively simple mix design was adopted for the porous concrete surface layer. The surface layer comprised nine distinct mix proportions, formulated by combining three single-sized aggregates (2.36–4.75 mm, 4.75–9.5 mm, and 9.5–13.2 mm) with three different cement-to-aggregate (C/A) ratios (0.22, 0.24, and 0.26), while maintaining a constant water-to-cement (W/C) ratio of 0.30. The specific mix designs are presented in Table 3.

Due to the dimensional constraints of the standing wave tube testing apparatus, the porous concrete specimens were fabricated as cylindrical samples with a diameter of 100 mm and a thickness of 50 mm. Specimen preparation followed the relevant Chinese industry standards CJJ/T 135–2009^36^. After casting, the specimens underwent a 1 d moist curing process in their molds, during which they were water-sprayed and covered. Subsequent curing was performed outdoors with regular water spraying until the specimens reached 28 d of age, followed by oven drying and cooling to room temperature prior to sound absorption testing. For each mix proportion, five replicate specimens were prepared, and the reported results represent the average of these replicates.

**Table 3:** Mixture proportions of porous concrete surface layer.

Specimen type	Aggregate gradation (content)			C/A	W/C	Measured porosity (%)
	2.36–4.75 mm(%)	4.75–9.5 mm(%)	9.5–13.2 mm(%)			
PS1-1*	100	0	0	0.22	0.30	22.5
PS1-2				0.24		17.3
PS1-3				0.26		13.9
PS2-1	0	100	0	0.22		20.4
PS2-2				0.24		16.4
PS2-3				0.26		14.4
PS3-1	0	0	100	0.22		22.4
PS3-2				0.24		20.3
PS3-3				0.26		16.1

* In subsequent text, specimen PS1-1 refers to a porous concrete mixture prepared with 2.36–4.75 mm aggregates, a cement-to-aggregate ratio of 0.22, and a water-to-cement ratio of 0.30. Other specimens follow the same naming convention (e.g., PS1-2, PS2-1, etc.), with alphanumeric codes corresponding to their specific mix parameters as defined in Table 3.

### Crushed stone base course

In this study, nine combinations of crushed stone base courses were examined, comprising three uniformly graded aggregate sizes (2.36–4.75 mm, 4.75–9.5 mm, and 9.5–13.2 mm) and three thickness levels (4 cm, 7 cm, and 10 cm) in the crushed stone base course, as detailed in Table 4.

**Table 4:** Design schemes for crushed stone base course.

Specimen ID	Crushed stone base course gradation (content)			Thickness (cm)
	2.36–4.75 mm(%)	4.75–9.5 mm (%)	9.5–13.2 mm(%)	
B0S1*	100			0
B0S2		100		
B0S3			100	
B4S1	100			4
B4S2		100		
B4S3			100	
B7S1	100			7
B7S2		100		
B7S3			100	
B10S1	100			10
B10S2		100		
B10S3			100	

*In subsequent text, specimens BS1, BS2, and BS3 denote crushed stone base courses with aggregate particle sizes of 2.36–4.75 mm, 4.75–9.5 mm, and 9.5–13.2 mm, respectively. Specimens B4S1, B7S1, and B10S1 represent crushed stone base courses with thicknesses of 4 cm, 7 cm, and 10 cm, respectively, all prepared using the 2.36–4.75 mm aggregate (BS1). Other specimens in Table 4 follow the same naming convention.

### Porosity

The porosity of the porous concrete specimens was determined using the underwater weighing method, with five replicate specimens tested. The specimens were cylindrical, measuring 100 mm in diameter and 50 mm in height. The porosity (P) was calculated using Eq. (1)^36^:

1 $$p=\left[1-\left( \frac{(m_1-m_2)/ \rho_w} {v} \right) \right] \times 100\%$$

Where *P* is the measured porosity of the porous concrete specimen; *m*_*1*_ is the saturated weight of the specimen in air, g; *m*_*2*_ is the saturated weight of the specimen in water, g; *ρ*_*w*_ is the density of water, taken as 1.0 g/cm^3^; *v* is the volume of the specimen, taken as 392.5 cm^3^ in this research. The average porosity of the five specimens was reported as the sample’s measured porosity.

### Sound absorption property

**Fig. 1:**
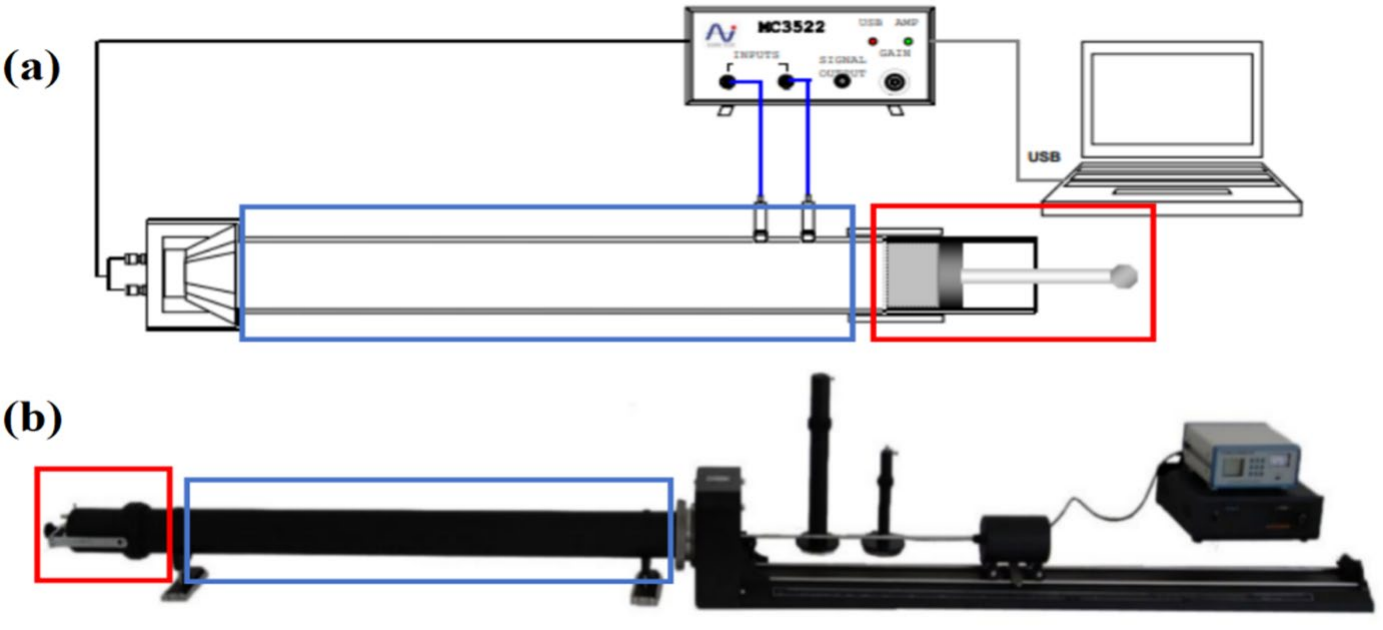
Standing wave tube sound absorption testing system; (a)Schematic diagram, (b) Actual photo.

The sound absorption property of porous concrete was assessed using a standing wave tube in this study, as shown in Fig. 1. For sound absorption testing of the single-layer porous concrete, a specimen with a thickness of 50 mm and diameter of 100 mm was placed in the low-frequency tube of the standing wave tube test system (as indicated in the red box in Fig. 1a; inner diameter: 100 mm). The piston of the low-frequency tube was then adjusted to align the test surface of the porous concrete specimen flush with the tube opening. The assembly was fixed to the long probe tube (shown in the blue box in Fig. 1a; inner diameter:100 mm), and parameters were configured to evaluate the noise reduction performance. The frequency-sound absorption coefficient curve within the 200–2000 Hz range was subsequently obtained. For the composite pavament structure, the piston in the low-frequency tube was first positioned such that the distance from the piston top to the tube opening matched the total design thickness (e.g. 9 cm for a 4 cm crushed stone base plus a 5 cm porous concrete layer). The low-frequency tube was then oriented vertically. Without mving the piston, crushed stone of varying particle sizes was carefully added to simulate the base layer, while the vertical distance from the top of the stone layer to the tube opening was continuously monitored until it reached 5 cm. The porous concrete surface layer was then inserted, ensuring its surface was flush with the tube opening. Finally, the low-frequency tube was carefully rotated to a horizontal position and fixed to the long probe tube for noise reduction testing. The frequency-sound absorption coefficient curve within the 200–2000 Hz range was similarly recorded for the composite structure.

Sound absorption tests of porous concrete were conducted across 11 frequencies (200, 250, 315, 400, 500, 630, 800, 1000, 1600, and 2000 Hz), and the mean absorption coefficient was adopted as the primary metric for evaluating noise reduction. Each test group consisted of five replicate specimens, and the average value was taken as the group’s sound absorption performance. The sound absorption coefficient (α) was calculated using Eq. (2)^3^:

2 $$\alpha =\frac{\alpha_1 + \cdots + \alpha_n}{\rm n}$$

Where *α* is the full-spectrum mean sound absorption coefficient; *α*_*1*_ is the sound absorption coefficient at 200 Hz; *α*_*n*_ is the sound absorption coefficient at 2000 Hz; *n* is the total number of tested frequencies (in this study, *n* = 11).

## Results and discussion

This section presents the sound absorption results of the porous concrete surface layer and the composite pavement structure. The effects of aggregate size, cement-to-aggregate ratio, and porosity of the surface layer was first examined. Then the influence of the crushed stone base, including its aggregate size and thickness was analyzed. Finally, the different surface layer types in the composite system was compared. These results help identify the main factors that control sound absorption in the 200–2000 Hz range.

### Impact of mix proportion on the sound absorption characteristics of porous concrete surfacing

This section examines how mix proportions affect the sound absorption of the surface layer.

#### Impact of aggregate size on the sound absorption property of porous concrete surfacing

This subsection analyzes the effect of aggregate size on the surface layer’s sound absorption.

**Fig. 2:**
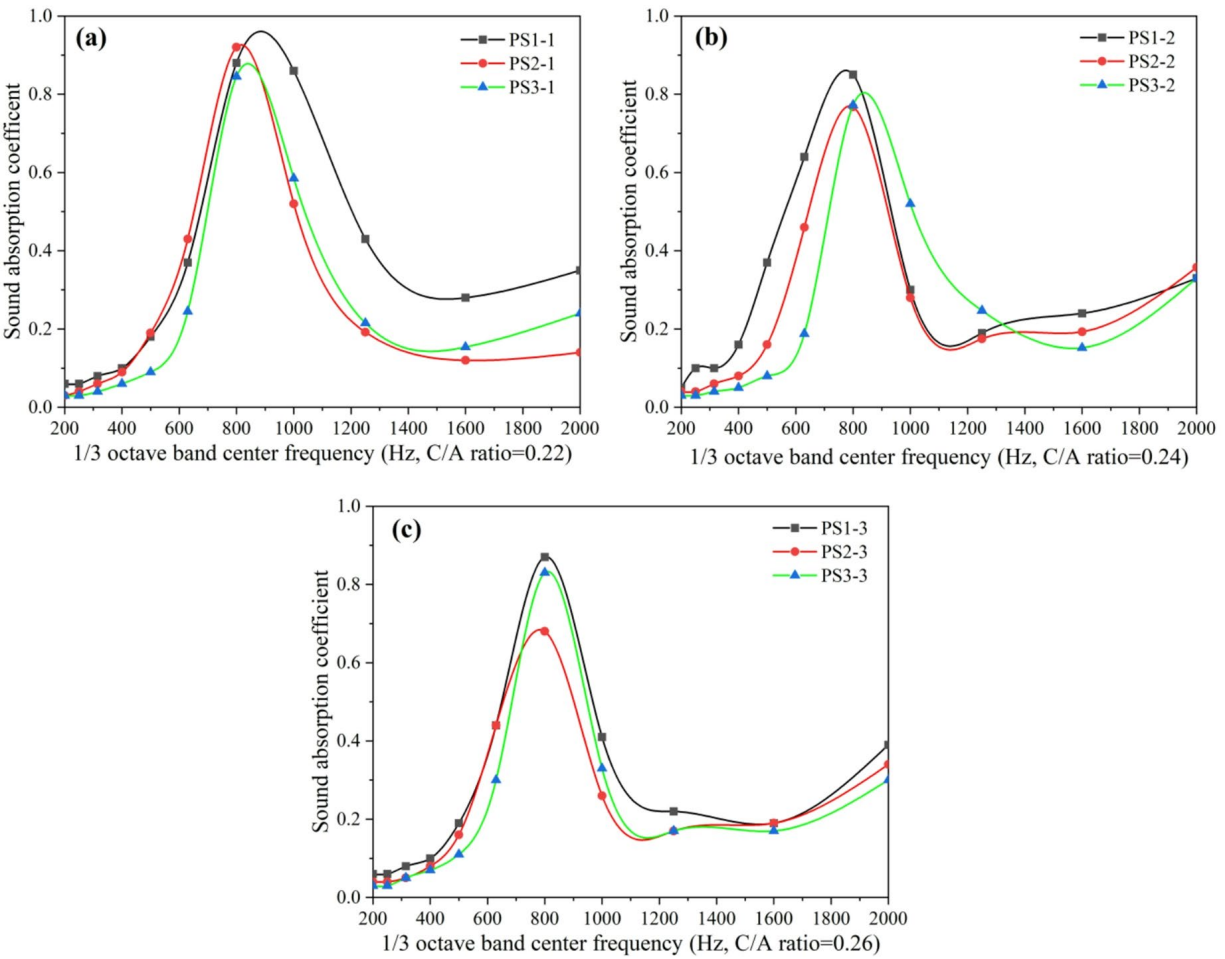
Effect of aggregate size on the sound absorption spectrum of porous concrete pavement; (a) C/A ratio = 0.22, (b) C/A ratio = 0.24, (c) C/A ratio = 0.26.

Figure 2 illustrates the effect of aggregate size on the sound absorption spectrum of porous concrete pavement under constant cement-to-aggregate ratio (C/A) and water-to-cement (W/C) ratio. When other conditions were held constant, all three porous concrete specimens with different aggregate sizes (5 cm thickness) exhibited a consistent trend in their sound absorption coefficient curves: an initial increase with frequency (200–800 Hz), a subsequent decrease (800–1600 Hz), and a final rise (1600–2000 Hz). The curves peaked at 800 Hz, with absorption coefficients exceeding 0.68 and reaching up to 0.92. These results indicate that variations in aggregate size do not affect the overall absorption trend or the dominant absorption frequency of porous concrete. However, 5 cm-thick specimens across all aggregate sizes showed relatively poor absorption in the 200–500 Hz range, while exhibiting significantly better performance in the 500–1250 Hz range. This frequency dependent behavior suggests that the wavelength of incident sound waves influence the effectiveness of porous concrete in sound absorption.

**Fig. 3:**
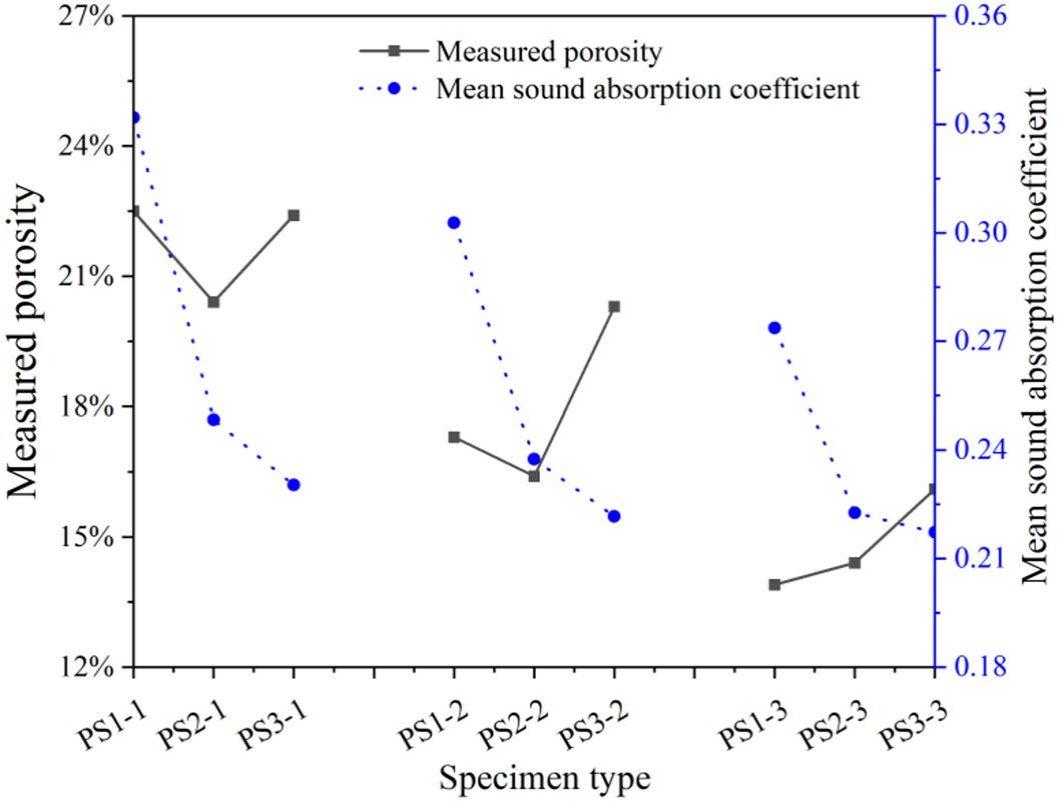
Effect of aggregate size on the sound absorption performance of porous concrete surfaces.

Figure 3 presents the influence of aggregate size on the sound absorption performance of porous concrete pavement. When maintaining identical C/A ratio, W/C ratio, and specimen thickness, the sound absorption capability exhibited a clear decreasing trend with increasing aggregate size, indicating that smaller aggregates enhance sound performance. Notably, no significant positive or negative correlation was observed between sound absorption performance and measured porosity across different aggregate sizes. This suggests that porosity alone cannot fully predict the sound absorption behavior of porous concrete; other micro-structural features, such as pore number, connectivity, tortuosity, and surface morphology, likely play an important role in the sound attenuation mechanisms.

#### Impact of cement-to-aggregate ratio on sound absorption of porous concrete surfacing

This subsection examines how the cement-to-aggregate ratio influences sound absorption.

**Fig. 4:**
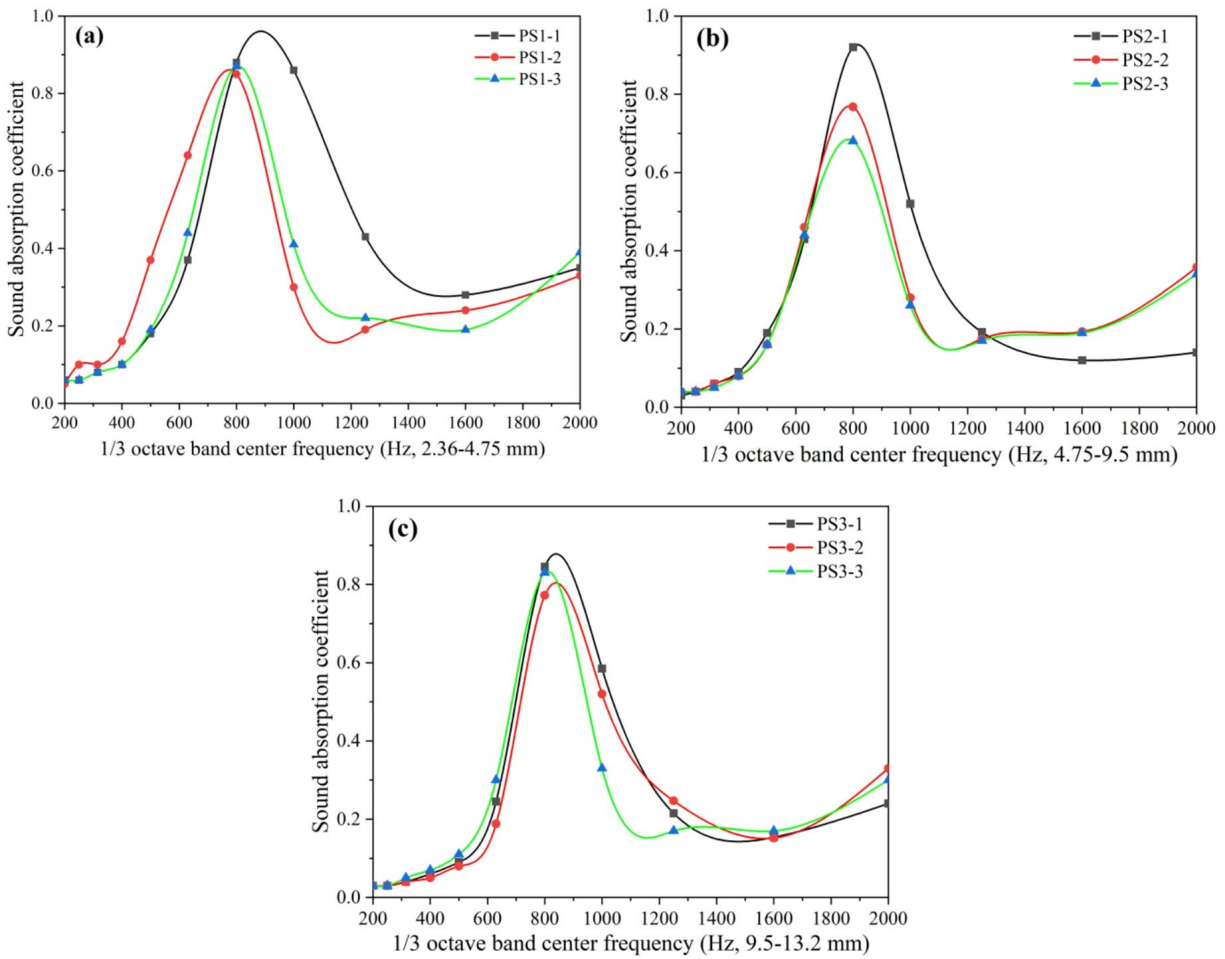
Effect of C/A ratio on the sound absorption spectrum of porous concrete pavement; (a) aggregate size 2.36–4.75 mm, (b) aggregate size 4.75–9.5 mm, (c) aggregate size 9.5–13.2 mm.

The influence of the C/A ratio on the sound absorption coefficient of the porous concrete surface layer is presented in Fig. 4. When aggregate size, W/C ratio, and specimen thickness were held constant, the sound absorption coefficient curves for different C/A ratios exhibited trends similar to those observed for different aggregate sizes. All curves showed an initial increase, followed by a decrease and a subsequent rise, with a single peak occurring in the entire frequency range. This indicates that the C/A ratio does not affect the overall sound absorption trend or the dominant absorption frequency. However, the C/A ratio does influence absorption effectiveness at specific frequencies, particularly in the 600–1250 Hz range.

**Fig. 5:**
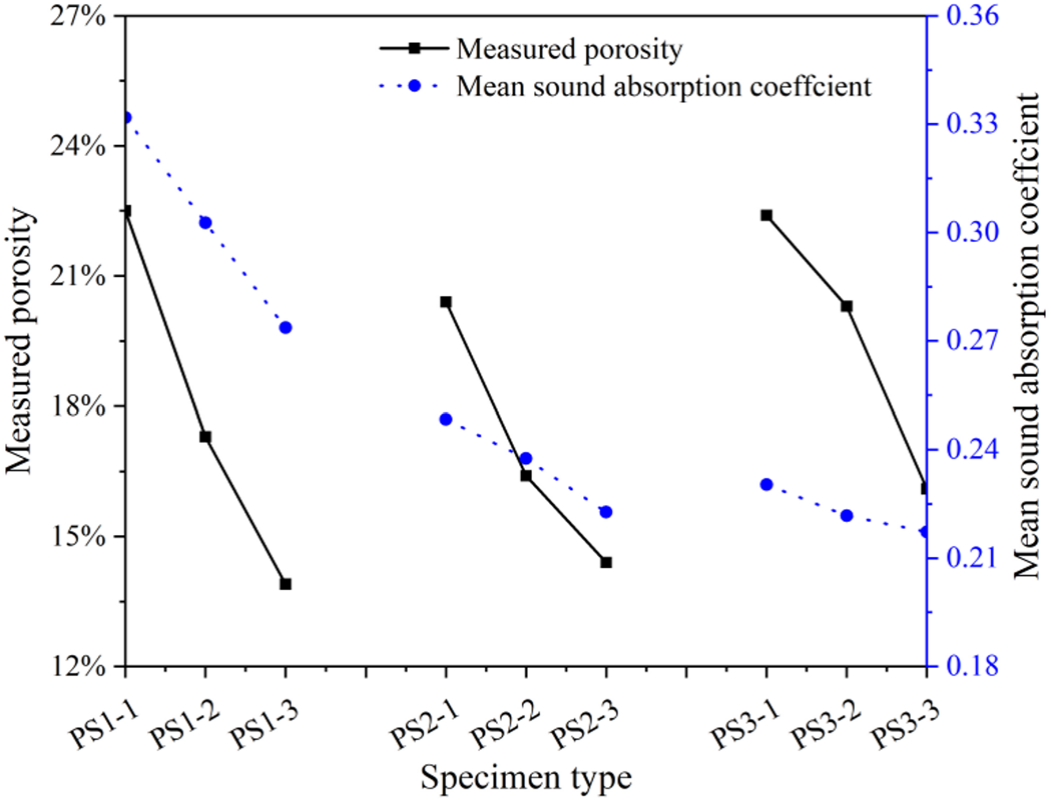
Effect of C/A ratio on the sound absorption performance of porous concrete pavement surfaces.

The influence of the C/A ratio on the sound absorption performance of porous concrete pavement is shown in Fig. 5. When aggregate size, W/C ratio, and specimen thickness were held constant, sound absorption decreased as the C/A ratio increased, indicating that lower C/A ratios enhance acoustic performance. This improvement is attributed to the increased porosity associated with lower C/A ratios, which allows greater penetration of sound waves into the internal structure. Higher porosity facilitates energy dissipation and absorption, thereby improving the material’s sound absorption. Furthermore, a significant positive correlation was observed between sound absorption and specimen porosity across different C/A ratios under otherwise identical conditions. These results suggest that the C/A ratio and the resulting porosity can serve as a primary indicator for evaluating the sound performance of porous concrete when other parameters are kept constant.

#### Correlation between measured porosity and sound absorption performance of porous concrete surfacing

This subsection explores the relationship between porosity and sound absorption property.

**Fig. 6:**
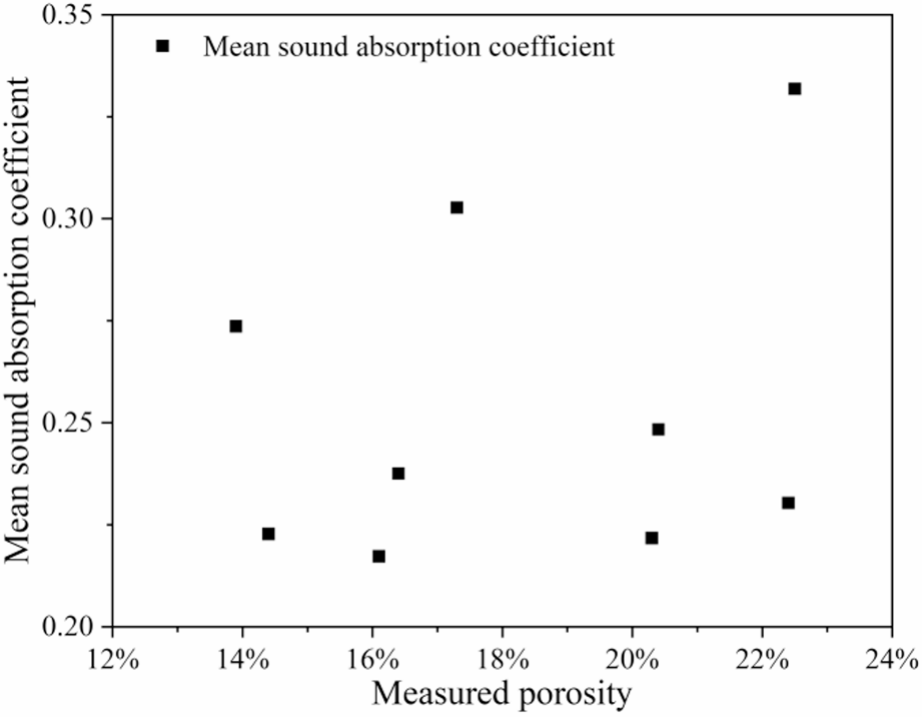
Correlation between porosity and sound absorption property of porous concrete pavement surfaces.

The relationship between sound absorption performance and measured porosity was analyzed for nine porous concrete specimens with varying aggregate sizes and C/A ratios, as shown in Fig. 6. The results demonstrate that, despite variations in aggregate size and C/A ratio, no significant correlation was observed between porosity and sound absorption performance. Therefore, for porous concrete prepared with different aggregate gradations and C/A ratios, porosity alone cannot be used as a reliable indicator of sound absorption performance.

### Effect of crushed stone base aggregate particle size on sound absorption of porous concrete pavement structure

 This section studies how the aggregate size of the crushed stone base affects the composite pavement’s sound absorption.And the sound absorption of the composite pavement was measured using the procedure described in Sect. 2.5. The crushed stone base was first filled into the standing wave tube to the required thickness, and the porous concrete surface layer was then placed on top. The tube was rotated to the horizontal position and connected before testing. This setup was used for all samples in this Sect. 3.2 to Sect. 3.4.

**Fig. 7:**
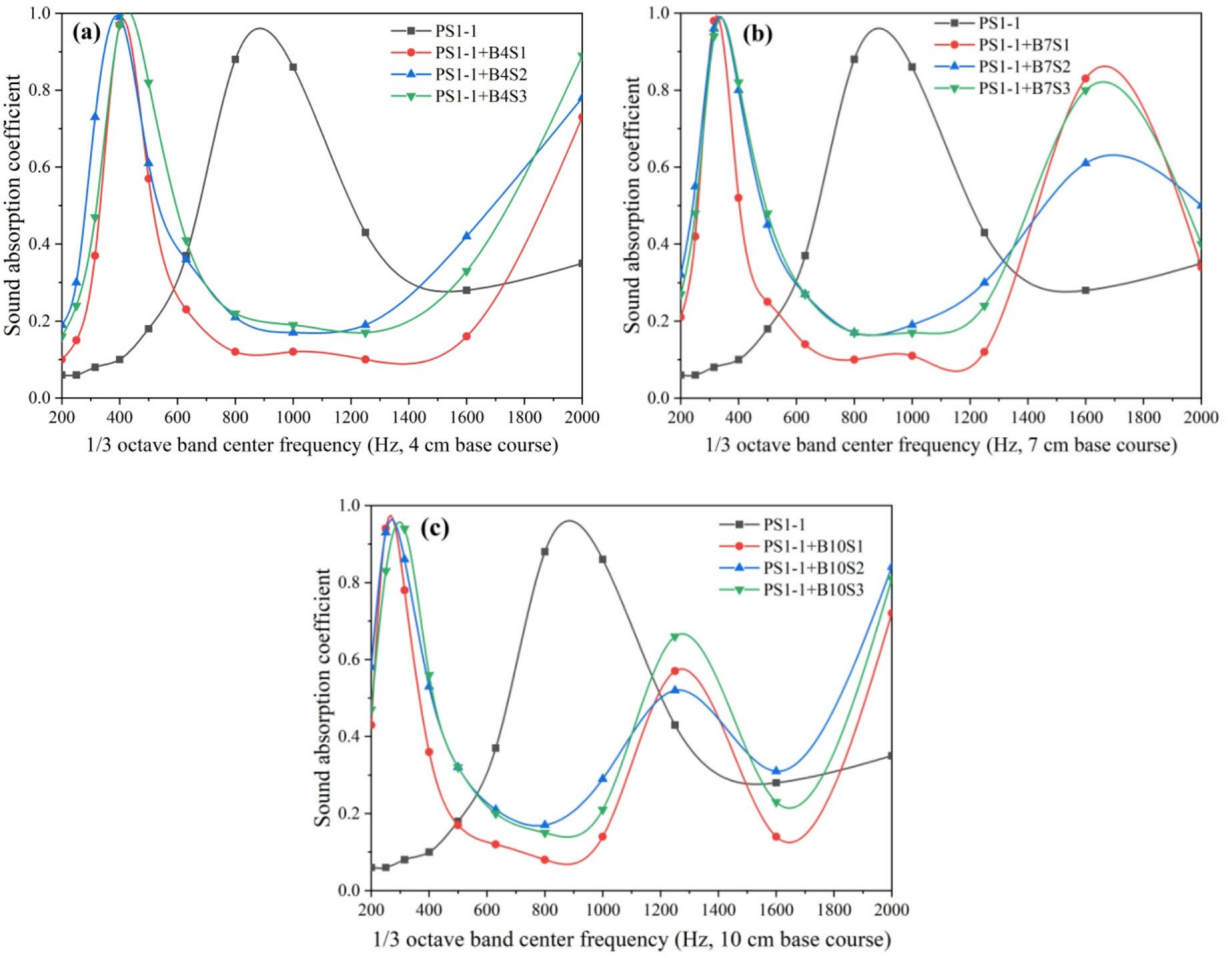
Effects of crushed stone base layer particle size on sound absorption coefficient curves of porous concrete pavement structures; (**a**) 4 cm base course layer, (**b**) 7 cm base course layer, (**c**) 10 cm base course layer.

Using the porous concrete pavement structure (comprising a wearing course PS1-1 and nine crushed stone base layers with varying aggregate sizes and thicknesses) as an example, this study examined the influence of base course aggregate gradation on sound absorption across different frequencies, as shown in Fig. 7. The results reveal the followings: (1) For crushed stone base layers with thicknesses of 4 cm, 7 cm, and 10 cm, the sound absorption coefficient curves of the corresponding pavement structures exhibit 1, 2, and 2 peaks, respectively, corresponding to 1, 2, and 2 dominant absorption frequencies. The dominant absorption frequencies occur at 400 Hz (4 cm), 315 Hz, 1600 Hz (7 cm), 250 Hz (with a secondary peak at 315 Hz), and 1250 Hz (10 cm). (2) Compared with the pavement structure consisting only of the surface layer, incorporation of a crushed stone base shifts the first dominant absorption frequency from higher to lower values. Furthermore, increasing base thickness leads to a further reduction in dominant absorption frequencies. This indicates that the crushed stone base affects not only the frequencies of dominant absorption peaks but also the number of peaks, depending on its thickness. (3) Under the same conditions, changes in aggregate size of the crushed stone base do not alter the frequencies or general pattern of absorption peaks; however, they do influence absorption performance within the 200–2000 Hz range.

**Fig. 8:**
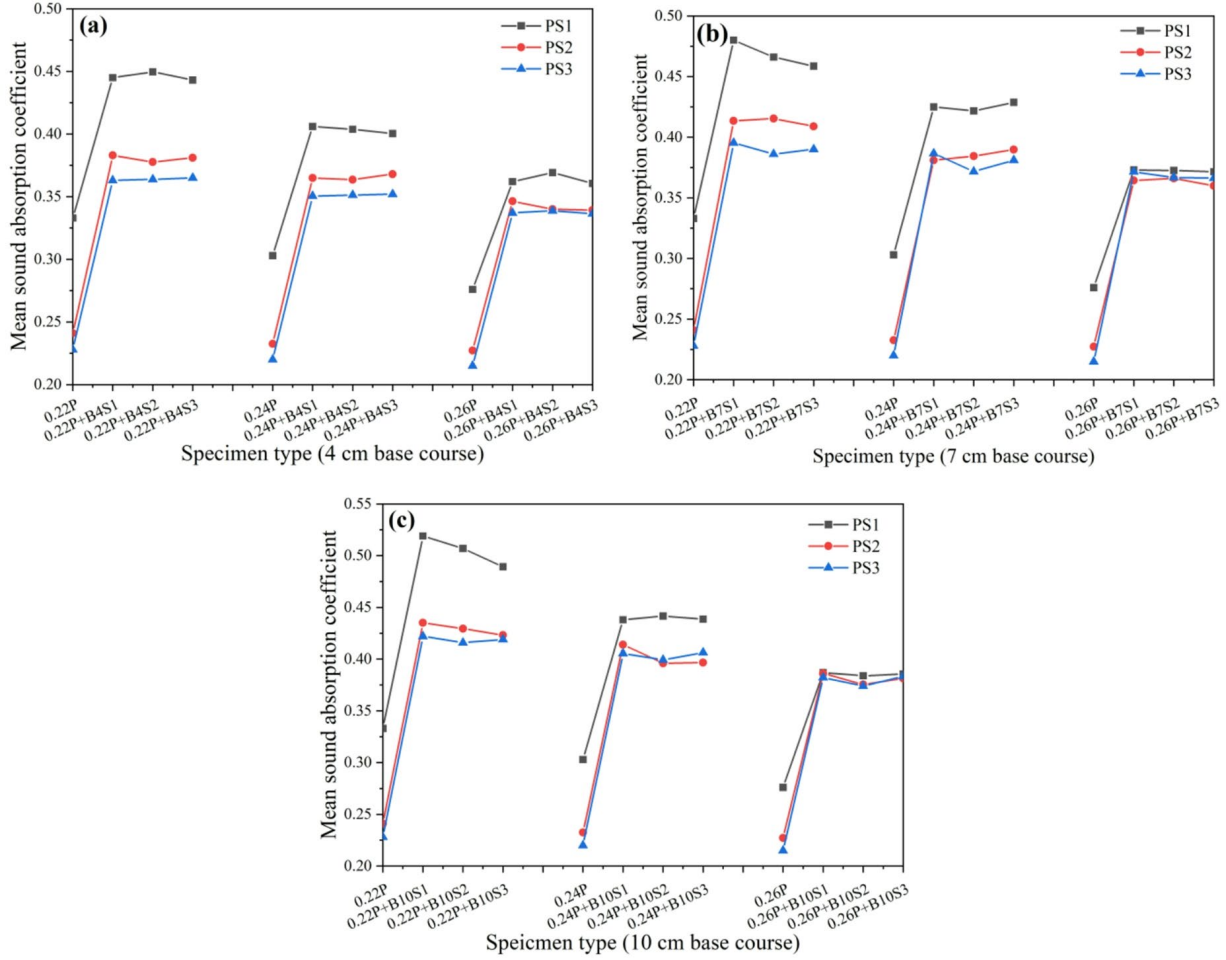
Effects of crushed stone base layer particle size on sound absorption performance of porous concrete pavement structures*; (**a**) 4 cm base course layer, (**b**) 7 cm base course layer, (**c**) 10 cm base course layer.

The influence of crushed stone base aggregate size on the sound absorption performance of porous concrete pavement structures is presented in Fig. 8. The results show that pavement structures incorporating a crushed stone base layer exhibit significantly better sound absorption performance than those consisting only of a wearing course. The crushed stone base can be regarded as a porous concrete structural layer with porosity approximately equal to the packed void ratio of the aggregates. When combined with the wearing course, it forms a dual-layer pavement system comprising a smaller-porosity upper layer and a larger-porosity lower layer, where the increased overall thickness enhances the sound absorption capability. Aggregate particle size in the crushed stone base significantly affects the sound absorption performance of the composite pavement structure; however, its influence depends on the aggregate size of the wearing course and the thickness of the base layer. Specifically, when the wearing course contains 2.36–4.75 mm aggregates with cement-to-aggregate (C/A) ratios of 0.22 and 0.24, sound absorption performance is markedly higher than that of surfaces with other aggregate sizes under the same conditions. This performance advantage diminishes at a C/A ratio of 0.26, where all three aggregate sizes yield comparable results. Under otherwise identical conditions, pavement structures with different crushed stone base aggregate sizes show similar overall sound absorption performance, suggesting that base aggregate size has only a limited influence. This outcome may be attributed to the loose state of the base aggregates, where porosity remains consistently high (41.6%−42.3%) regardless of particle size.

*The labels 0.22P, 0.24P, and 0.26P in Fig. 8 denote the C/A ratio (0.22, 0.24, and 0.26, respectively) in the porous concrete wearing course.

### Effect of crushed stone base course thickness on sound absorption of porous concrete pavement structure

This section evaluates the influence of base thickness on the sound absorption of the composite pavement.

**Fig. 9:**
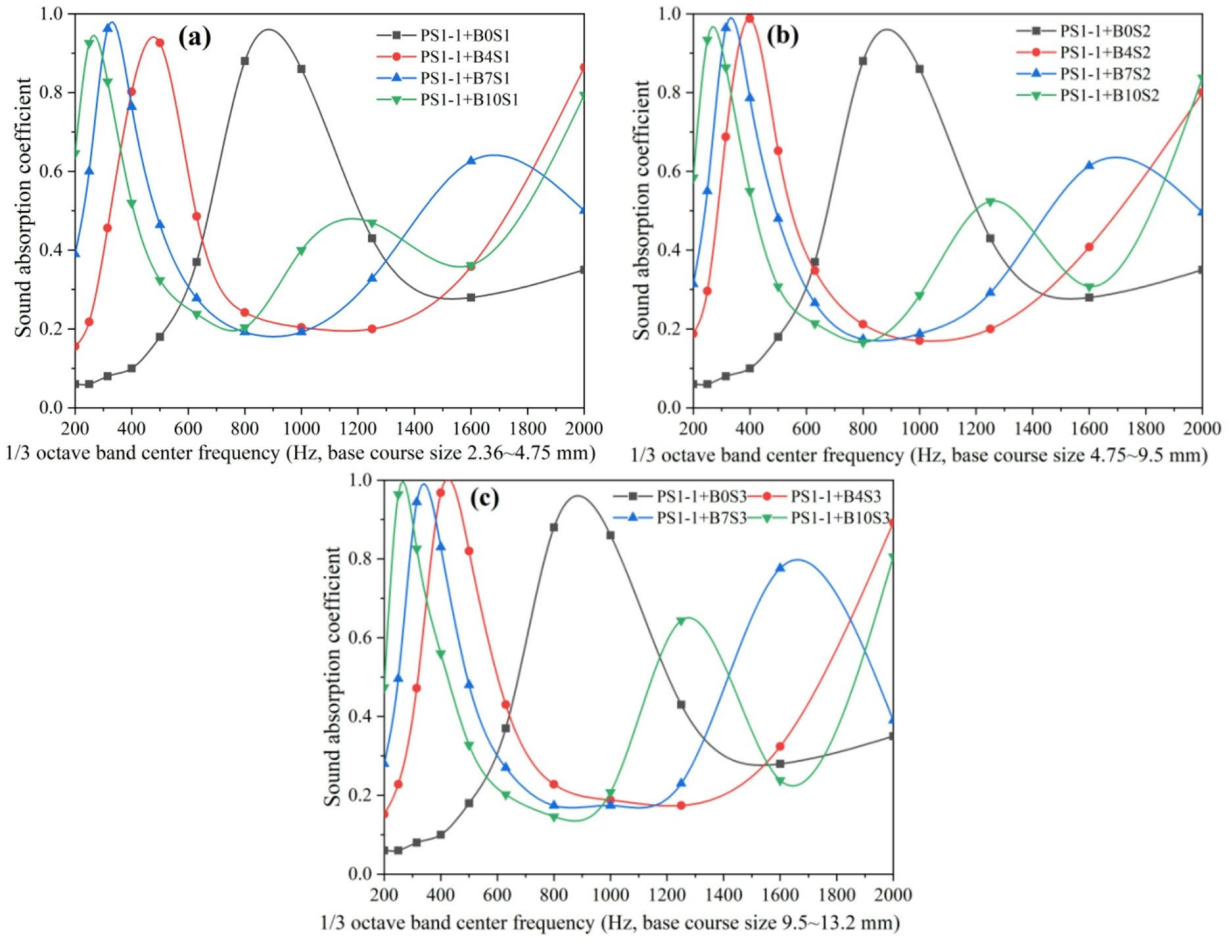
Influence of crushed stone base layer thickness on sound absorption coefficient curve of porous concrete pavement structures: (**a**) base course layer size 2.36–4.75 mm, (**b**) base course layer size 4.75–9.5 mm, (**c**) base course layer size 9.5–13.2 mm.

The influence of crushed stone base layer thickness on the sound absorption performance of porous concrete pavement structures (comprising a wearing surface PS1-1 with 2.36–4.75 mm aggregates, a cement-to-aggregate ratio of 0.22, a water-to-cement ratio of 0.30, and a thickness of 5 cm, combined with nine crushed stone base configurations varying in aggregate size and thickness) is shown in Fig. 9. The results indicate: (1) For crushed stone base thicknesses of 0 cm, 4 cm, 7 cm, and 10 cm, the first dominant sound absorption frequencies consistently occurred at 800 Hz, 400 Hz, 350 Hz, and 250 Hz, respectively, regardless of aggregate size. This demonstrates that increasing base thickness shifts the first absorption peak from higher to lower frequencies, while aggregate size has a negligible effect on peak frequency position. When base thicknesses were 0 cm and 4 cm, single absorption peaks were observed at 800 Hz and 315 Hz, respectively. At thicknesses of 7 cm and 10 cm, dual absorption peaks appeared at 315 Hz and 1600 Hz (7 cm) and at 250 Hz and 1250 Hz (10 cm). These results suggest that base thickness influences both the number of absorption peaks and their frequency distribution, with secondary peaks also shifting downward as thickness increases. (2) For pavement structures with base thicknesses of 4 cm, 7 cm, and 10 cm, variations in base aggregate size significantly affected sound absorption performance across frequencies when the wearing surface contained 2.36–4.75 mm aggregates. For example, at a base thickness of 7 cm, the sound absorption coefficients at the second peak frequency (1600 Hz) were 0.61 for 2.36–4.75 mm aggregates, 0.61 for 4.75–9.5 mm aggregates, and 0.78 for 9.5–13.2 mm aggregates, representing a 28% improvement.

**Fig. 10:**
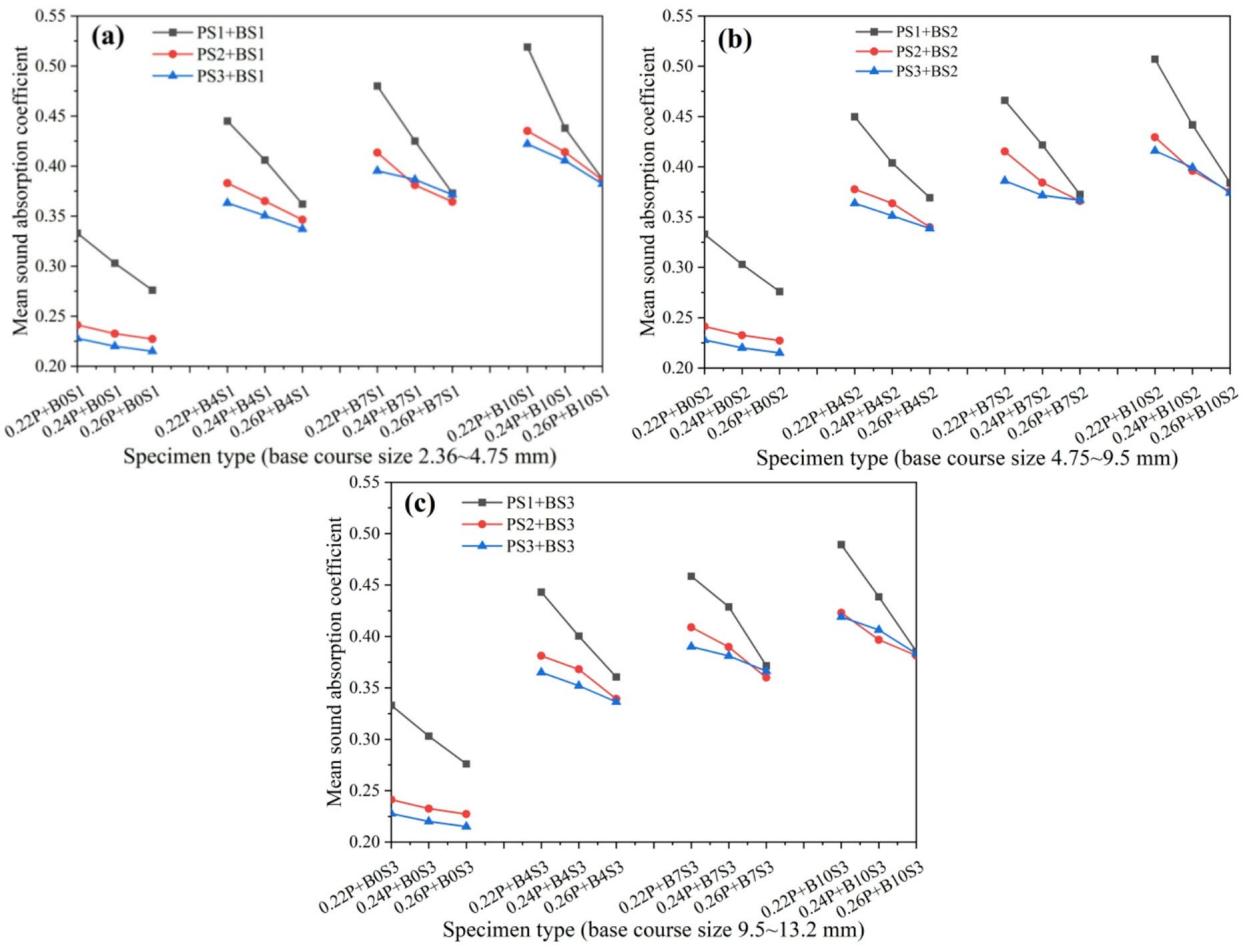
Effect of crushed stone base layer thickness on the sound absorption performance of porous concrete pavement structures*; (**a**) base course layer size 2.36–4.75 mm, (**b**) base course layer size 4.75–9.5 mm, (**c**) base course layer size 9.5–13.2 mm.

The influence of crushed stone base thickness on the sound absorption performance of porous concrete pavement structures is shown in Fig. 10. The results show that the noise reduction performance of pavements with crushed stone bases consistently improves with increasing base thickness, indicating that greater thickness enhances the overall acoustic performance of the system. This suggests that increasing crushed stone base thickness is an effective approach for optimizing sound absorption. In contrast, no clear trend is observed with variations in crushed stone base aggregate size. When the surface material and base thickness are held constant, aggregate particle size has a negligible influence on the overall sound absorption performance of the pavement system.

* In the Fig. 10, PS1 + BS1, PS1 + BS2, and PS1 + BS3 represent porous concrete pavement structures composed of a surface layer with 2.36–4.75 mm aggregates and crushed stone base layers with aggregate sizes of 2.36–4.75 mm, 4.75–9.5 mm, and 9.5–13.2 mm, respectively, with all other parameters kept constant. Similarly, 0.22P + B4S1, 0.24P + B4S1, and 0.26P + B4S1 denote surface layers with cement-to-aggregate ratios of 0.22, 0.24, and 0.26, respectively, combined with a crushed stone base layer of 2.36–4.75 mm aggregate size and 4 cm thickness, under otherwise identical conditions.

### Effect of pavement surface type on sound absorption of porous concrete pavement structures

This section compares different surface layer types to assess their effect on the composite pavement’s sound absorption.

**Fig. 11:**
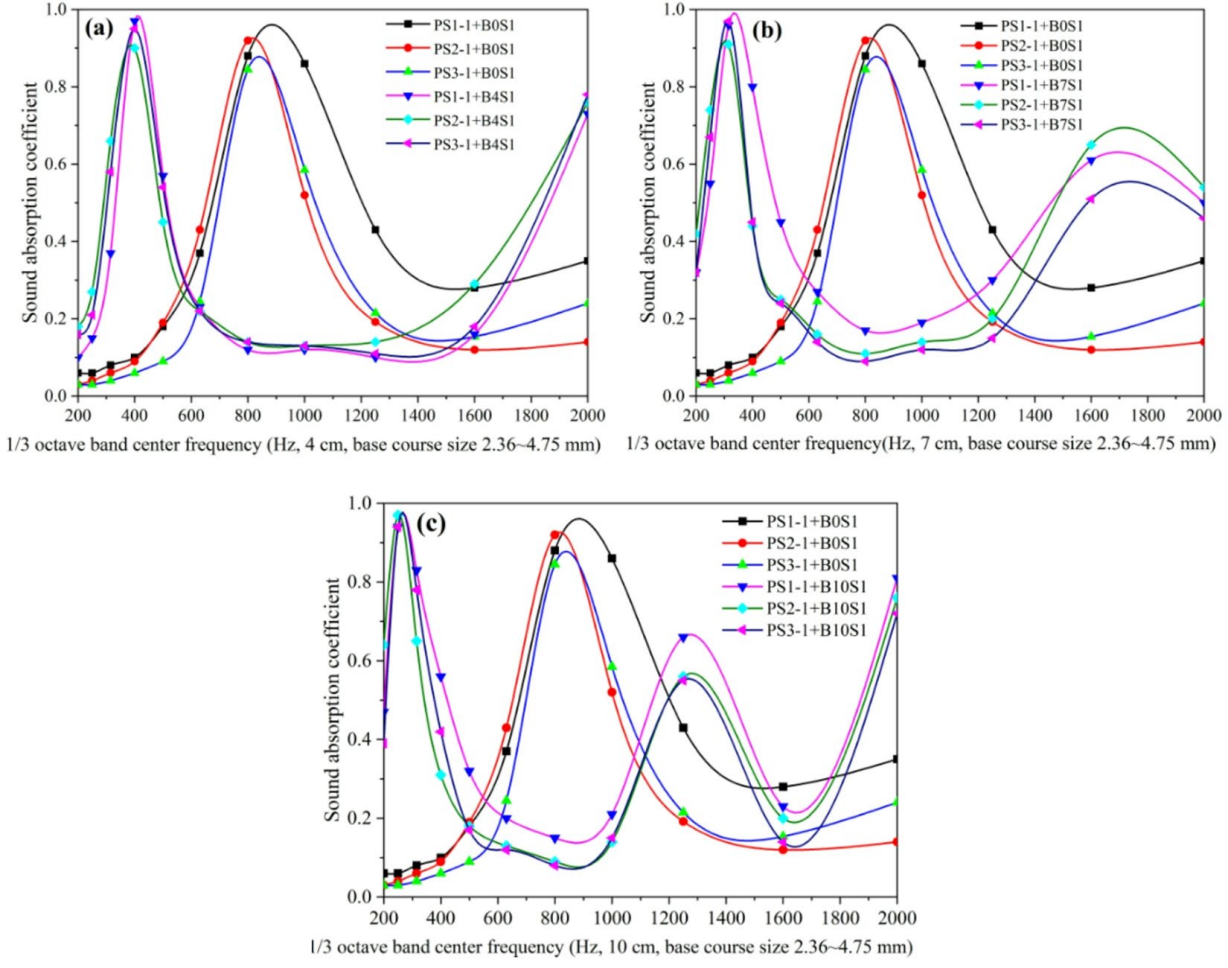
Effect of wearing course type on the sound absorption coefficient curve of porous concrete pavement structures: (**a**) 4 cm base course layer, (**b**) 7 cm base course layer, (**c**) 10 cm base course layer.

The effect of surface layer type on the sound absorption coefficient curves of porous concrete pavement structures with crushed stone bases was analyzed using surface layers (PS1-1, PS2-1, and PS3-1) with a C/A ratio of 0.22, W/C ratio of 0.30, and 5 cm thickness, combined with crushed stone bases of 4 cm, 7 cm, and 10 cm thickness (aggregate particle size: 2.36–4.75 mm). The results are shown in Fig. 11. Under identical conditions, the three porous concrete pavement surface layers with different aggregate sizes exhibited similar overall trends in their sound absorption coefficient curves. This suggests that the aggregate size of the porous concrete surface layer does not significantly influence the general absorption pattern across frequencies, although it may affect the absorption efficiency at specific frequencies. When the thickness of the crushed stone base layer was 4 cm, 7 cm, and 10 cm, the sound absorption coefficient curves exhibited 1, 2, and 2 peaks, respectively. This indicates that the base layer thickness governs the number of absorption peaks (i.e., dominant absorption frequencies), whereas the aggregate size of the surface layer does not alter the number of peaks. The dominant absorption frequencies of the porous concrete pavement structures with 4 cm, 7 cm, and 10 cm base thicknesses were 400 Hz, 315 Hz and 1600 Hz, and 250 Hz and 1250 Hz, respectively. These findings further confirm that the surface aggregate size does not affect the dominant absorption frequencies of the structure.

**Fig. 12:**
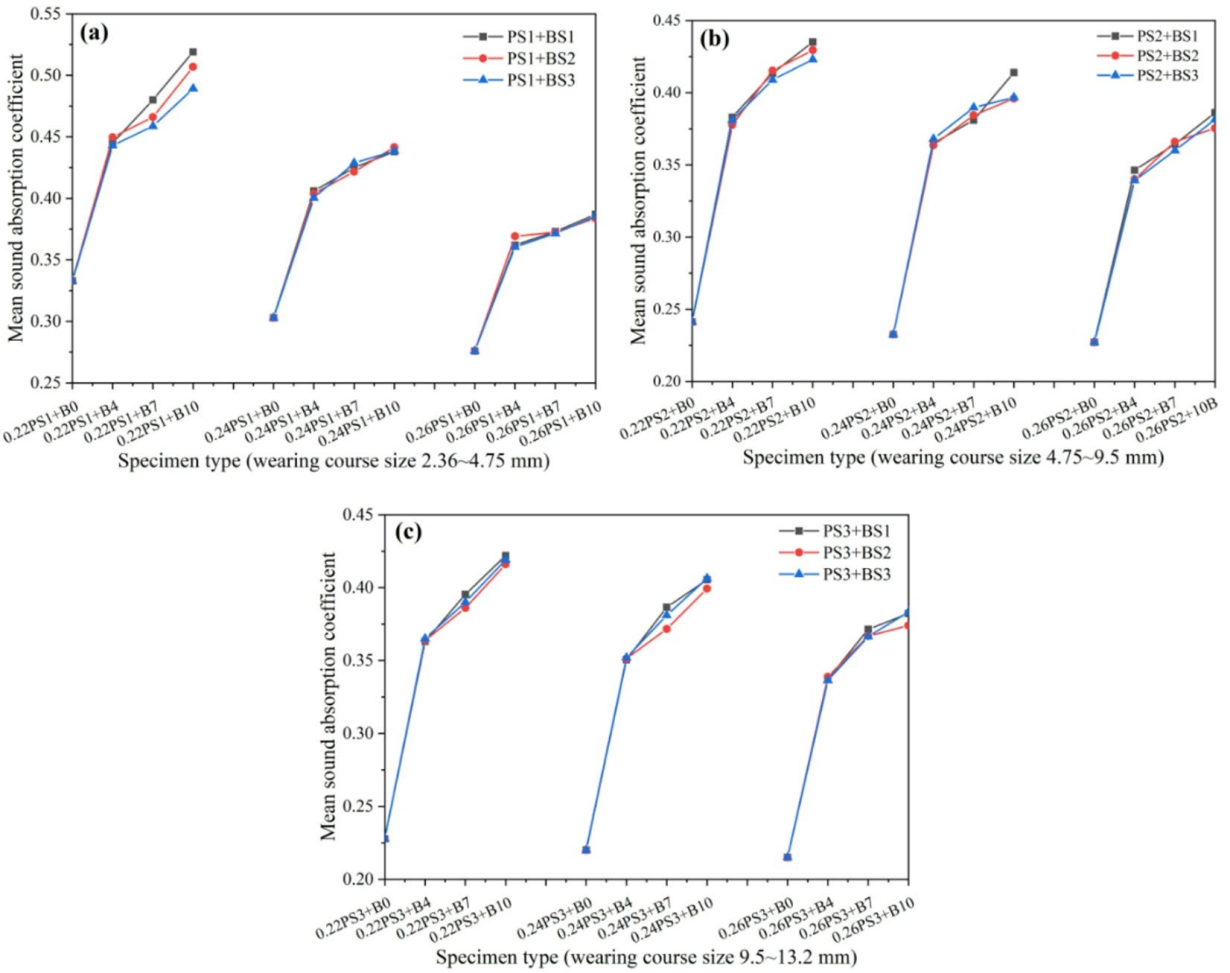
Influence of wearing course type on sound absorption properties of porous concrete pavement structures; (**a**) wearing course size 2.36–4.75 mm, (**b**) wearing course size 4.75–9.5 mm, (**c**) wearing course size 9.5–13.2 mm.

The results of the effect of pavement surface material type on the sound absorption performance of porous concrete pavement structures with a crushed stone base layer are presented in Fig. 12. When the aggregate size of the surface layer is 2.36–4.75 mm with cement-to-aggregate (C/A) ratios of 0.22 and 0.24, the sound absorption performance of the pavement structure is significantly higher than that of structures with larger aggregate sizes of 4.75–9.5 mm and 9.5–13.2 mm. The latter two show comparable performance, particularly when the C/A ratio is 0.26, where their sound absorption curves are nearly identical. When the aggregate size is 2.36–4.75 mm with a C/A ratio of 0.26, the sound absorption performance remains superior to that of structures with larger aggregate sizes; however, the difference is less pronounced compared with lower C/A ratios. Under otherwise identical conditions, the overall sound absorption performance of porous concrete pavement structures decreases with increasing C/A ratio of the surface layer material. This trend is primarily attributed to the reduction in porosity at higher C/A ratios, which decreases the likelihood of sound waves penetrating into the pavement structure. Consequently, less sound energy is dissipated internally, while more is reflected or refracted back to the receiver, leading to diminished noise reduction. Therefore, reducing the C/A ratio of the surface layer material (i.e., increasing porosity) can effectively enhance the sound absorption capability of porous concrete pavement structures.

## Conclusion

This study was conducted to determine how both the surface layer and the crushed stone base contribute to the sound absorption performance of porous concrete pavement structures within the 200–2000 Hz range. The main findings are as follows::

1. Aggregate size and C/A ratio affect the absorption level but do not change the curve shape or peak frequency. Smaller aggregates improve absorption by about 10–20%, and lower C/A ratios increase absorption by about 8–15%. These results are consistent with previous studies on porous concrete.
2. Adding a crushed stone base shifts the dominant absorption frequency to a lower range and increases overall absorption. Increasing the base thickness from 0 cm to 10 cm raises the mean absorption coefficient by roughly 20–30%, which agrees with earlier research showing that thicker porous layers enhance low-frequency performance.
3. The aggregate size of the crushed stone base has only a small influence on absorption, which is similar to reports that high-void aggregates show limited changes with size.
4. Using smaller aggregates and lower C/A ratios in the surface layer improves the absorption of the composite pavement, while the base thickness controls the number and position of peak frequencies.

These findings offer guidance for designing porous concrete pavements with better acoustic performance. Future work may include field testing and more evaluation indices to further validate the results.

## Data Availability

All data generated or analyzed during this study are included in this published article.
